# High-Throughput Quantitative Proteomic Analysis of Dengue Virus Type 2 Infected A549 Cells

**DOI:** 10.1371/journal.pone.0093305

**Published:** 2014-03-26

**Authors:** Han-Chen Chiu, Holger Hannemann, Kate J. Heesom, David A. Matthews, Andrew D. Davidson

**Affiliations:** 1 School of Cellular and Molecular Medicine Faculty of Medical and Veterinary Sciences, University of Bristol, Bristol, United Kingdom; 2 Proteomics Facility, Faculty of Medical and Veterinary Sciences, University of Bristol, Bristol, United Kingdom; Kantonal Hospital St. Gallen, Switzerland

## Abstract

Disease caused by dengue virus is a global health concern with up to 390 million individuals infected annually worldwide. There are no vaccines or antiviral compounds available to either prevent or treat dengue disease which may be fatal. To increase our understanding of the interaction of dengue virus with the host cell, we analyzed changes in the proteome of human A549 cells in response to dengue virus type 2 infection using stable isotope labelling in cell culture (SILAC) in combination with high-throughput mass spectrometry (MS). Mock and infected A549 cells were fractionated into nuclear and cytoplasmic extracts before analysis to identify proteins that redistribute between cellular compartments during infection and reduce the complexity of the analysis. We identified and quantified 3098 and 2115 proteins in the cytoplasmic and nuclear fractions respectively. Proteins that showed a significant alteration in amount during infection were examined using gene enrichment, pathway and network analysis tools. The analyses revealed that dengue virus infection modulated the amounts of proteins involved in the interferon and unfolded protein responses, lipid metabolism and the cell cycle. The SILAC-MS results were validated for a select number of proteins over a time course of infection by Western blotting and immunofluorescence microscopy. Our study demonstrates for the first time the power of SILAC-MS for identifying and quantifying novel changes in cellular protein amounts in response to dengue virus infection.

## Introduction

The four serotypes of dengue virus (DENV types 1–4) cause the most important arthropod-borne viral disease of humans. DENV infection results in a range of clinical outcomes ranging from the milder dengue fever to the potentially life threatening dengue haemorrhagic fever/dengue shock syndrome [1]. A recent study estimates that up to 390 million people are infected with DENV annually [2], making dengue a serious global public-health problem. Despite much effort, there are neither vaccines nor antiviral therapies in clinical use to prevent or treat dengue, and our understanding of dengue pathogenesis is still limited.

DENV is a member of the *Flavivirus* genus of the *Flaviviridae* family and has a RNA genome of ∼11 kb in size. Translation of the genome results in the production of a single large polyprotein that is subsequently processed by a combination of cellular and the viral NS2B/3 proteinase to yield the three structural proteins capsid (C), pre-membrane (prM) and envelope (E) and the non-structural (NS) proteins, NS1, NS2A, NS2B, NS3, NS4A, 2K, NS4B and NS5 [3]. Replication of the DENV genome occurs in intimate association with perinuclear ER membranes which are modified to form characteristic structures during virus infection [4]. High-throughput RNA interference studies have shown that DENV depends heavily on the cellular machinery for replication [5], [6]. However the mechanisms by which DENV interacts with cellular pathways and the viral and cellular proteins involved, largely remain to be determined.

Comparative analysis of the gene expression profiles of a range of cell types infected with DENV *in vitro* [7]–[13] and cells isolated from the blood of DENV infected individuals [14]–[19] has identified a number of genes and cellular signaling pathways that are specifically dysregulated in DENV infection and may be involved in pathogenesis. In addition, high-throughput interaction studies [20]–[22] have identified a number of interactions between DENV and cellular proteins that may play a role in replication or avoiding host defense mechanisms.

By contrast to gene expression studies, the analysis of the host response to either DENV or flavivirus infection at the proteomic level is more limited. The standard approach of two-dimensional (2D) PAGE combined with the identification of specific proteins by mass spectrometry (MS) has been used to detect proteins that are altered in amount in DENV infected mammalian cells [23]–[27], insect cells [28], [29] and in sera from DENV infected patients [30], [31] and resulted in the identification of a number of cellular proteins potentially relevant to pathogenesis. However this type of analysis is limited by the resolution and sensitivity of 2D-PAGE.

In recent years, advances in the sensitivity of MS, coupled with high-throughput protein identification has made it feasible to quantify global changes in cellular protein levels in response to viral infection. The use of stable isotope labeling techniques to distinguish proteins derived from different cell populations, either by metabolic labeling of proteins (stable isotope labeling by amino acids in cell culture; SILAC) or chemical modification of peptides (ie tandem mass tagging), in combination with quantitative MS, provides the most sensitive means of accurately analyzing the proteome of a cell currently available. By combining differential labeling techniques with subcellular fractionation and quantitative MS, it is possible not only to measure changes in the amounts of proteins, but also to study changes in the cellular distribution of proteins, even if the total protein levels have not altered significantly [32], [33]. This approach is well suited to the comparative analysis of cell populations such as control and virus infected cells, but surprisingly there are very few reports of the application of these techniques to study viral pathogenesis [34] and none for DENV.

In this study we investigated the effects of DENV-2 infection on the host cell proteome of human A549 cells using SILAC in combination with high throughput liquid chromatography (LC)-MS/MS. The mock and infected A549 cells were fractionated into nuclear and cytoplasmic extracts before analysis to identify proteins that redistribute between cellular compartments during infection and reduce the complexity of the analysis. We identified proteins that both increased and decreased in response to DENV-2 infection, including many novel proteins not previously identified to be effected by DENV infection. Bioinformatic analysis was used to identify a number of processes affected by DENV-2 infection. The results of the SILAC-MS analysis was validated for seven selected proteins by Western blotting and immunofluorescence microscopy. This is the first report of the application of SILAC-MS to study changes in the host cell proteome in response to DENV infection and demonstrates the power of this technique for identifying and quantifying changes in cellular protein amounts in response to DENV infection.

## Materials and Methods

### Cell lines and viruses

Human lung carcinoma (A549; ATCC CCL-185) and human epithelial kidney cells (HEK293; ATCC CCL-1573) were cultured in Dulbecco's modified Eagle's medium (DMEM) with glutamax (Invitrogen) supplemented with 0.1 nM non-essential amino acids and 10% foetal bovine serum (FBS) (Invitrogen). All cells were maintained at 37 °C and 5% CO_2_ in a humidified atmosphere. DENV-2 strain New Guinea C (GenBank accession number: AF038403) was propagated and titered, and cells infected with DENV-2 as described previously [35], [36].

### SILAC labeling of cells, DENV-2 infection and cellular fractionation

For the SILAC analysis, A549 cells were grown in DMEM containing either unlabeled arginine and lysine amino acids (R0K0) or ^13^C labeled arginine and lysine amino acids (R6K6) (Dundee Cell Products, UK) supplemented with 10% SILAC dialyzed FBS (MWCO 10,000 Da, Dundee Cell Products). After 8 population doublings, 4×10^7^ A549 cells grown in the R6K6 media were infected with DENV-2 at a multiplicity of infection (m.o.i.) of 5 whilst 4×10^7^ A549 cells grown in the R0K0 media were mock infected using previously described infection conditions [36]. At 28 hours post infection (p.i.) the culture supernatants were removed, the cells were washed twice with ice cold phosphate buffered saline (PBS), detached and harvested by centrifugation. The cell pellets were resuspended in swelling buffer (10 mM Hepes, pH 7.9, 10 mM KCl, 1.5 mM MgCl_2_, 0.5 mM DTT) and incubated on ice for 10 min. The cell membrane was then disrupted using a dounce homogenizer. The cell lysates were centrifuged at 250 *g* for 5 min at 4 °C. The cytoplasmic fractions were removed, added to an equal volume of 2X SDS-PAGE sample buffer and heated at 95 °C for 10 min. The nuclear pellets were resuspended in 3 ml of buffer S1 (0.25 M sucrose, 10 mM MgCl_2_), layered over a 3 ml cushion of buffer S2 (0.35 M sucrose, 0.5 mM MgCl_2_) and centrifuged at 1500 *g* for 5 min at 4°C. The supernatant was removed and the nuclear pellet resuspended in 200 μl of buffer S2 followed by disruption of the nuclei by sonication (3×20 sec) using a Bioruptor (Diagenode, Belgium). The protein concentration in each fraction was determined using a BCA Protein Assay kit (Pierce - Thermo Scientific). Twenty μg of protein from the cytoplasmic fraction prepared from the DENV-2 infected and mock infected cells were mixed and the process repeated for the nuclear fractions. The proteins in the two samples were then separated by one-dimensional SDS-PAGE and stained using Coomassie blue. Each of the lanes was excised and used for LC-MS/MS analysis.

### LC-MS/MS analysis

Each gel lane was cut into 10 slices and each slice subjected to in-gel tryptic digestion using a ProGest automated digestion unit (Digilab, UK). The resulting peptides were fractionated using a Dionex Ultimate 3000 nanoHPLC system in line with an LTQ-Orbitrap Velos mass spectrometer (Thermo Scientific). In brief, peptides in 1% (v/v) formic acid were injected onto an Acclaim PepMap C18 nano-trap column (Dionex). After washing with 0.5% (v/v) acetonitrile 0.1% (v/v) formic acid, peptides were resolved on a 250 mm×75 μm Acclaim PepMap C18 reverse phase analytical column (Dionex) over a 150 min organic gradient, using 7 gradient segments (1–6% solvent B over 1 min, 6–15% B over 58 min, 15–32% B over 58 min, 32–40% B over 3 min, 40–90% B over 1 min, held at 90% B for 6 min and then reduced to 1% B over 1 min) with a flow rate of 300 nl min^−1^. Solvent A was 0.1% formic acid and Solvent B was aqueous 80% acetonitrile in 0.1% formic acid. Peptides were ionized by nano-electrospray ionization at 2.3 kV using a stainless steel emitter with an internal diameter of 30 μm (Thermo Scientific) and a capillary temperature of 250 °C. Tandem mass spectra were acquired using an LTQ-Orbitrap Velos mass spectrometer controlled by Xcalibur 2.1 software (Thermo Scientific) and operated in data-dependent acquisition mode. The Orbitrap was set to analyze the survey scans at 60,000 resolution (at m/z 400) in the mass range m/z 300 to 2000 and the top six multiply charged ions in each duty cycle selected for MS/MS in the LTQ linear ion trap. Charge state filtering, where unassigned precursor ions were not selected for fragmentation, and dynamic exclusion (repeat count, 1; repeat duration, 30 sec; exclusion list size, 500) were used. Fragmentation conditions in the LTQ were as follows: normalized collision energy, 40%; activation q, 0.25; activation time 10 msec; and minimum ion selection intensity, 500 counts.

### Quantification and bioinformatic analysis

The raw data files were processed and quantified using MaxQuant (version 1.2.2.5) [37]. The Andromeda search engine [38] was used to search the MS/MS spectra against the UniProt/SwissProt human database release version 57.3 (20326 entries) and a FASTA file containing the DENV-2 New Guinea C strain (GenBank accession number: AF038403) polyprotein and individual processed proteins. Cysteine carbamidomethylation was set as a fixed modification and methionine oxidation, N-terminal acetylation and the SILAC labels (^13^C-lysine, ^13^C-arginine) as variable modifications in the search. Searches were performed with full tryptic digestion, a MS tolerance of 6 ppm, a maximum number of 5 modifications per peptide and a minimum peptide length of 6, a maximum of 2 missed cleavages and a maximum charge of 7. Reverse database search options were enabled and contaminants included. The MS/MS tolerance was set at 0.5 Da and the false discovery rate (FDR) for peptides and proteins was set to 0.01. Protein quantification was done using razor and unique peptides and protein ratios were calculated as the median of the raw measured peptide ratios for each protein. Only proteins with two or more peptide quantification ratios were used for bioinformatic analysis. A posterior error probability (PEP) score was generated for each protein. Only proteins with a PEP of less than 0.1 were considered in the analysis. Proteins that were found to be increased or decreased by ≥1.5 fold in DENV-2 infected cells were analyzed using the Software Tool for Researching Annotations of Proteins (STRAP) [39], the Database for Annotation, Visualization and Integrated Discovery (DAVID) v 6.7 [40], [41] and the Search Tool for the Retrieval of Interacting Genes/Proteins (STRING) 9.1 database [42] to identify classes of proteins belonging to specific processes and pathways that were over-represented in DENV infected cells.

### Western blotting and antibodies

The protein concentration in cell lysates was measured using a BCA Protein Assay kit (Pierce - Thermo Scientific), separated by 10% SDS-PAGE and then transferred from the gel to a polyvinylidene difluoride membrane (GE Healthcare Life Sciences) using a Trans-Blot Semi-Dry Transfer Cell (Bio-Rad). The membrane was blocked in TBST (50 mM Tris-HCl pH 7.6, 150 mM NaCl, 0.05% (v/v) Tween) containing 5% w/v skim milk powder for 1 hour before incubation with an appropriate primary antibody diluted in the blocking solution for 1 hour at room temperature. The following primary antibodies were used anti-PRAF2, anti-HYOU1, anti-ERC1 (ab53113, ab124884 and ab50312 from Abcam), anti-β-tubulin (2146S, New England Biolabs), anti-lamin A/C, anti-KPNA2, anti-UBE2S, anti-CTSL1, anti-GAPDH (SAB4200236, I1784, SAB2102626, SAB4500559 and G8795 from Sigma-Aldrich) and anti-MFN1 (sc-50330 from Santa Cruz). After washing with TBST, the membrane was incubated with appropriate dilutions of anti-mouse IgG (12–349, Millipore) or anti-rabbit IgG (sc-2054, Santa Cruz) secondary antibodies conjugated to horseradish peroxidise in blocking solution for 1 hour at room temperature. Following further washes in TBST, proteins were detected by enhanced chemiluminescence using a LumiGLO Chemiluminescent Substrate (Kirkegaard & Perry Laboratories) followed by exposure to X-ray film (Amersham Hyperfilm ECL, GE Healthcare Limited).

### Immunofluorescence assay and confocal microscopy

Cells grown on glass cover slips in 24 well trays were either infected with DENV-2 or mock infected. The cells were fixed with 4% formaldehyde in PBS for 5 min followed by permeabilization in 1% (v/v) Triton X-100 and then analyzed by immunofluorescence assay (IFA) as previously described using a mixture of anti-DENV E or NS5 protein antibodies [43] to detect DENV-2 infected cells and primary antibodies against the ERC1 and PRAF2 proteins and secondary antibodies coupled to Alexa-Fluor 568 or Alexa-Fluor 488 (Molecular Probes, Invitrogen). Cells were mounted in Vectashield containing DAPI (Vecta Laboratories). Confocal laser scanning microscopy was done using a Leica confocal microscope (TCS-SP2). The images were processed using the Volocity 6.0 software package (Perkin-Elmer). The images shown are a typical result from at least two independent experiments.

## Results and Discussion

### Infection and fractionation of DENV infected cells

**Figure 1:**
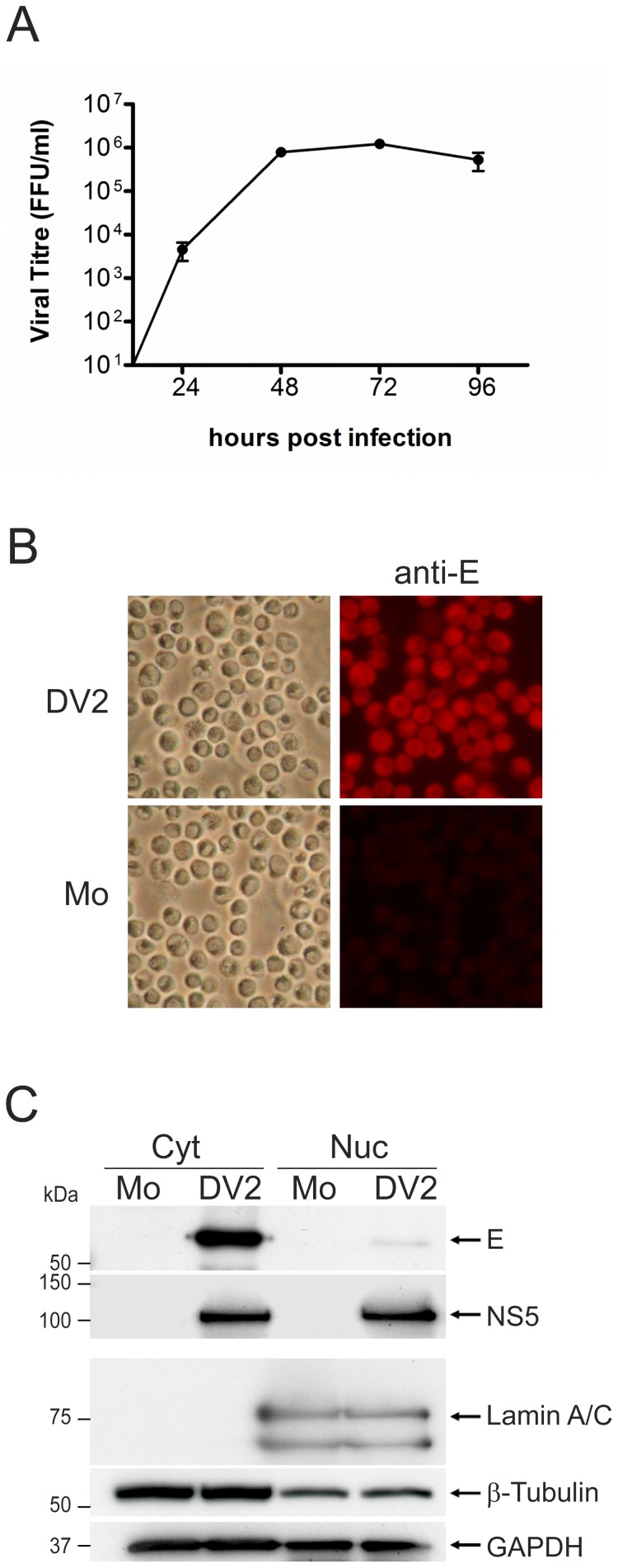
Confirmation of the viral infection and cellular fractionation procedures prior to LC-MS/MS analysis. (A) A549 cells were infected with DENV-2 at a m.o.i. of 5. Samples of the culture supernatant were taken over the period 24–96 hours post-infection and assayed for the presence of infectious virus by immunofocus assay. The titers are shown as focus forming units (FFU) per ml and are an average of two independent experiments with error bars shown. (B) SILAC labeled A549 cells that had been either DENV-2 (DV2) or mock (Mo) infected at a m.o.i. of 5 were detached with trypsin at 28 hours p.i. and harvested prior to fractionation. Samples of the detached cells were applied to glass coverslips, fixed with methanol and examined for the presence of the DENV-2 E protein by immunostaining using a specific antibody (anti-E) (right-hand panels) or observed under the light microscope (left-hand panels). (B) Western blot analysis of cytoplasmic (Cyt) and nuclear (Nuc) fractions prepared from the SILAC labeled A549 cells infected with DENV-2 or mock infected. Ten μg of protein from each cellular fraction was analyzed for the presence of the DENV-2 E and NS5 proteins and the cellular proteins lamin A/C, β-tubulin and GAPDH using specific anti-sera. The results shown are typical of two independent Western blotting experiments. The positions of relevant molecular mass markers are shown in kDa.

In order to conduct the proteomic analysis, human lung carcinoma A549 cells were grown for eight cell doublings in either light (R0K0) or heavy (R6K6) labeled media before being mock infected or infected with DENV-2 respectively. Although not believed to represent a target cell for DENV *in vivo*, A549 cells are highly permissive for DENV infection and have been used in previous studies examining the effect of DENV on the cellular innate immune response [44], [45] and the host cell transcriptome [8]. At 28 hours p.i., a time point determined by growth curve analysis (Figure 1A) to lie in exponential phase of DENV replication, the cells were harvested and fractionated into nuclear and cytoplasmic extracts. At this time there was no obvious difference in the morphology of the mock and DENV infected cells, suggesting that there was little cell death (data not shown). IFA analysis of a sample of the mock and infected A549 cells revealed that 100% of the cells had been infected (Figure 1B). The fractionation was done to reduce the overall complexity of the sample and to identify proteins that were altered in amount or redistributed between the cytoplasm and nucleus during DENV infection. DENV is known to replicate in tight association with perinuclear membranes [4] therefore the cells were fractionated using a procedure that removed as much of the perinuclear membrane as possible without disrupting the nuclei. The presence of protein markers in the cellular fractions specific to the nucleus, cytoplasm and DENV infection were analyzed by Western blotting to validate the infection and fractionation procedures (Figure 1C). The analysis showed that whilst the nuclear and soluble cytoplasmic protein fractions were distinct, the lack of detergent in the lysis buffer led to the presence of some membraneous/cytoskeletal proteins in the nuclear fractions. It appeared that the perinuclear membrane and the associated viral replication structures were not totally removed from the nuclei, as evidenced by a minor amount of the virus E protein, which is cytoplasmically localized, in the nuclear fraction. By contrast, the NS5 protein is known to be found in both the nucleus and cytoplasm of infected cells [43].

### Quantitative LC-MS/MS analysis

**Figure 2:**
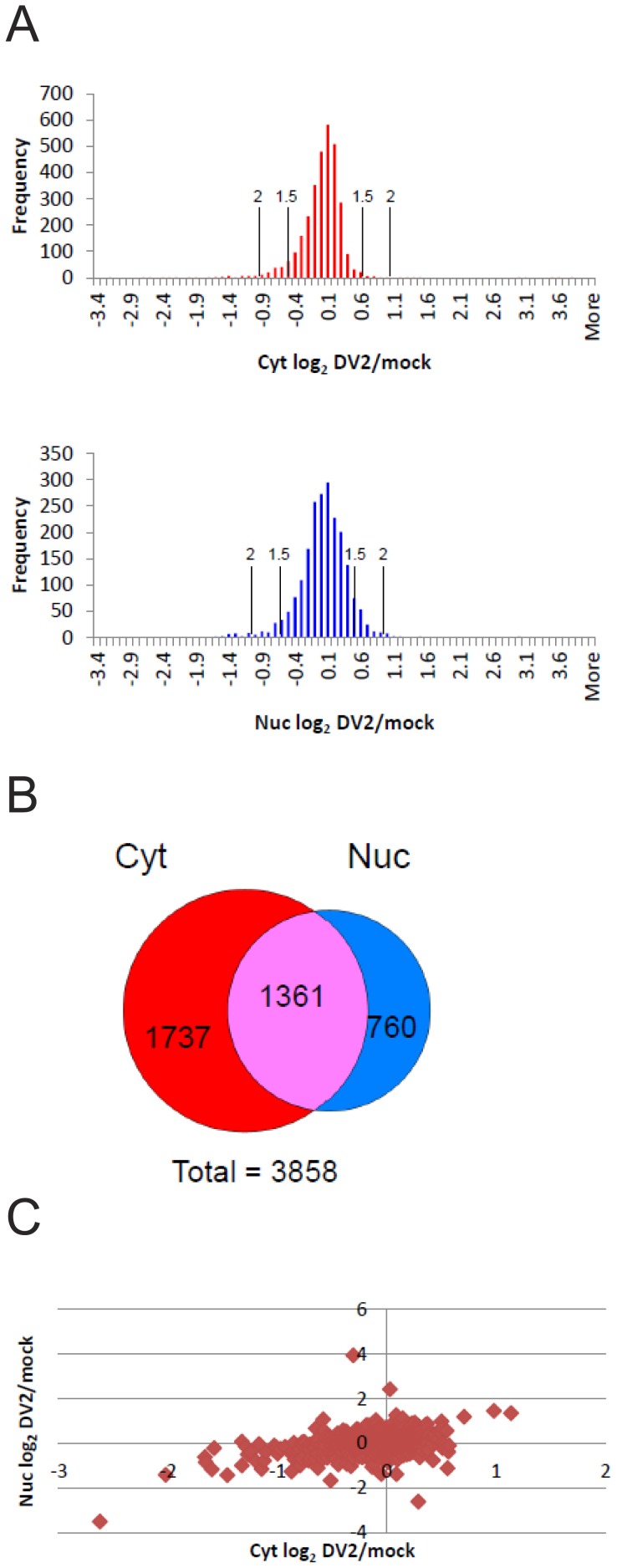
Results of the SILAC-MS analysis. (A) Histograms showing the frequency of the SILAC log_2_ ratios of proteins in cytoplasmic (top panel) and nuclear (bottom panel) fractions from DENV-2 (DV2) and mock infected cells. The log_2_ ratios were grouped into bins. The number of ratios per bin is shown on the *y*-axis. The cut-off points for 1.5 and 2 fold (log_2_ values of ± 0.585 and 1 respectively) increases and decreases from the median values are shown. (B) A Venn diagram showing the number of proteins that were detected either in both the cytoplasmic and nuclear fractions or exclusively in each fraction. (C) A scatter plot showing the nuclear (*y*-axis) and cytoplasmic (*x*-axis) SILAC log_2_ DV-2/mock ratios for the 1361 proteins that were identified and quantified in both fractions. The values for each protein are shown as a red diamond.

Equal protein amounts from the cytoplasmic fractions of the mock and DENV-2 infected cells were pooled and the same procedure repeated for the nuclear fractions. The proteins in the cytoplasmic and nuclear fractions were separated by 1D SDS-PAGE, subject to in-gel tryptic digestion and the peptides analyzed by quantitative LC-MS/MS to determine the relative amounts of proteins in the nuclear and cytoplasmic fractions from mock and DENV-2 infected cells. This procedure was done once and resulted in the identification of 4053 and 2881 cellular proteins in the cytoplasmic and nuclear fractions of which 3098 and 2115 respectively, were reliably quantified (based on the determination of a SILAC ratio for two or more peptides corresponding to each protein). Analysis of the distribution of log_2_ transformed SILAC ratios for the proteins in the cytoplasmic and nuclear extracts showed a symmetrical distribution around the normalized median value of 1 (log_2_ = 0) for both sets of proteins (Figure 2A) suggesting that there was no bias in the experimental approach used. There were 3858 proteins reliably quantified in total in both the nuclear and cytoplasmic fractions, of which 1361 proteins were common (Figure 2B). Typically, an alteration in protein amount between 1.3–2 fold has been considered significant [32]. In this analysis we used protein ratio cut-off values of 1.5 and 2 fold to select proteins for further bioinformatic analysis (as described below). The majority of both the cytoplasmic and nuclear proteins quantified remained relatively unchanged during DENV-2 infection with 94.5% and 90% of proteins in the cytoplasmic and nuclear fractions respectively showing a ≤1.5 fold change in amount (Figure 2A). In the cytoplasmic fraction 12 and 28 proteins showed 2 or 1.5 fold increases and 57 and 183 proteins showed 2 or 1.5 fold decreases respectively in DENV-2 infected cells compared to mock infected cells (Table 1 and Table S1). By comparison, in the nuclear fraction, 13 and 82 proteins showed 2 or 1.5 fold increases and 44 and 125 proteins showed 2 or 1.5 fold decreases respectively in DENV-2 infected cells compared to the fraction from mock infected cells (Table 2 and Table S1).

**Table 1:** Cytoplasmic proteins increased or decreased in amount >2 fold during DENV-2 infection.

Gene Name^a^	UniprotID	Protein Name	DV2/Mock	Peps	PEP	Function
**Proteins increased >2fold in DENV-2 infected cells n = 12**						
IFIT3	O14879	Interferon-induced protein with tetratricopeptide repeats 3	13.66	5	1.62×10^−42^	IFN-induced antiviral protein which acts as an inhibitor of cellular as well as viral processes.
IFIT1	P09914	Interferon-induced protein with tetratricopeptide repeats 1	13.00	7	7.23×10^−31^	IFN-induced antiviral protein which inhibits expression of viral messenger RNAs lacking 2′-O-methylation of the 5′ cap
IFIT2	P09913	Interferon-induced protein with tetratricopeptide repeats 2	11.55	4	4.09×10^−23^	IFN-induced antiviral protein which inhibits expression of viral messenger RNAs lacking 2′-O-methylation of the 5′ cap
FAM3A	P98173	Protein FAM3A	10.96	2	2.70×10^−2^	May act as a defensin against invading fungal microorganisms.
ISG15	P05161	Ubiquitin-like protein ISG15	7.59	2	1.03×10^−7^	Ubiquitin-like protein that is conjugated to intracellular target proteins after IFN-alpha or IFN-beta stimulation.
FNTB	P49356	Protein farnesyltransferase subunit beta	2.67	2	6.33×10^−25^	Catalyzes the transfer of a farnesyl moiety from farnesyl pyrophosphate to a cysteine on target proteins.
ASC1P100	Q9H1I8	Activating signal cointegrator 1 complex subunit 2	2.52	2	1.24×10^−4^	Enhances NF-kappa-B, SRF and AP1 transactivation.
DDX58	O95786	Probable ATP-dependent RNA helicase DDX58	2.50	4	2.97×10^−25^	Innate immune receptor which acts as a cytoplasmic sensor of viral nucleic acids and activates a cascade of antiviral responses.
PRPF39	Q86UA1	Pre-mRNA-processing factor 39	2.33	2	4.17×10^−3^	Involved in pre-mRNA splicing
HSPB11	Q9Y547	Heat shock protein beta-11	2.24	3	2.51×10^−10^	Component of IFT complex B composed of IFT88, IFT57, TRAF3IP1, IFT52, IFT27, HSPB11 and IFT20.
SRPR	P08240	Signal recognition particle receptor subunit alpha	2.20	9	1.16×10^−10^	Component of the signal recognition particle receptor. Ensures the correct targeting of the nascent secretory proteins to the ER.
HSPA5	P11021	78 kDa glucose-regulated protein	1.98	101	0	Probably plays a role in facilitating the assembly of multimeric protein complexes inside the ER.
**Proteins decreased >2 fold in DENV-2 infected cells n = 57**						
ERC1	Q8IUD2	ELKS/Rab6-interacting/CAST family member 1	0.10	4	4.01×10^−20^	Regulatory subunit of the IKK complex. May be involved in Rab-6 regulated endosomes to Golgi transport.
KRT6B	P04259	Keratin, type II cytoskeletal 6B	0.11	8	5.12×10^−4^	Structural constituent of cytoskeleton
MOGS	Q13724	Mannosyl-oligosaccharide glucosidase	0.16	11	2.38×10^−17^	Cleaves the distal alpha 1,2-linked glucose residue from the Glc(3)Man(9)GlcNAc(2) oligosaccharide precursor.
BCAM	P50895	Basal cell adhesion molecule	0.19	4	2.75×10^−14^	Laminin alpha-5 receptor. May mediate intracellular signaling.
DDX3Y	O15523	ATP-dependent RNA helicase	0.20	2	1.52×10^−206^	Probable ATP-dependent RNA helicase. May play a role in spermatogenesis.
MMADHC	Q9H3L0	Methylmalonic aciduria and homocystinuria type D protein, mitochondrial	0.20	2	2.0×10^−3^	Involved in cobalamin metabolism.
MORF4L1	Q9UBU8	Mortality factor 4-like protein 1	0.24	4	1.64×10^−4^	Component of the NuA4 histone acetyltransferase complex which is involved in transcriptional activation of select genes.
AURKB	Q96GD4	Aurora kinase B	0.25	2	4.86×10^−3^	Serine/threonine-protein kinase component of the chromosomal passenger complex, a complex that acts as a regulator of mitosis.
DKFZp781G1976	Q5H9Q2	Putative uncharacterized protein DKFZp781G1976	0.26	2	2.72×10^−8^	Unknown
LAMC1	P11047	Laminin subunit gamma-1	0.30	2	1.95×10^−11^	Thought to mediate the attachment, migration and organization of cells into tissues during embryonic development.
NUCB1	Q02818	Nucleobindin-1	0.32	2	7.15×10^−22^	Major calcium-binding protein of the Golgi. May have a role in calcium homeostasis.
CHCHD2	Q9Y6H1	Coiled-coil-helix-coiled-coil-helix domain-containing protein 2, mitochondrial	0.32	2	2.11×10^−7^	Mitochondrial protein
NDUFS5	O43920	NADH dehydrogenase [ubiquinone] iron-sulfur protein 5	0.32	2	3.22×10^−9^	Accessory subunit of the mitochondrial membrane respiratory chain NADH dehydrogenase.
CLU	P10909	Clusterin	0.32	4	4.3×10^−8^	Isoform 1 functions as extracellular chaperone that prevents aggregation of nonnative proteins.I
ECT2	Q9H8V3	Protein ECT2	0.33	5	1.43×10^−7^	Guanine nucleotide exchange factor that catalyzes the exchange of GDP for GTP.
MGRN1	O60291	E3 ubiquitin-protein ligase MGRN1	0.33	2	1.09×10^−10^	E3 ubiquitin-protein ligase. Mediates monoubiquitination at multiple sites of TSG101 in the presence of UBE2D1.
PSAP	P07602	Proactivator polypeptide	0.34	10	1.25×10^−17^	Saposin-D is a specific sphingomyelin phosphodiesterase activator.
APP	P05067	Amyloid beta A4 protein	0.35	3	4.31×10^−13^	N-APP binds TNFRSF21 triggering caspase activation and degeneration of both neuronal cell bodies via caspase-3.
GRAMD1A	Q96CP6	GRAM domain-containing protein 1A	0.36	3	1.74×10^−5^	Single-pass membrane protein
SCD	O00767	Stearoyl–CoA desaturase	0.36	4	2.22×10^−5^	Component of the liver microsomal stearyl-CoA desaturase system, that catalyzes double bond formation into fatty acyl-CoA substrates.
NRCAM	Q92823	Neuronal cell adhesion molecule	0.37	5	1.73×10^−18^	Cell adhesion, ankyrin-binding protein involved in neuron-neuron adhesion.
P4HA2	O15460	Prolyl 4-hydroxylase subunit alpha-2	0.37	5	2.55×10^−9^	Catalyzes the post-translational formation of 4-hydroxyproline in -Xaa-Pro-Gly- sequences in collagens and other proteins.
PEG10	Q86TG7	Retrotransposon-derived protein PEG10	0.37	4	3.25×10^−11^	Prevents apoptosis in hepatocellular carcinoma (HCC) cells through interaction with SIAH1, a mediator of apoptosis.
CDK4	P11802	Cyclin-dependent kinase 4	0.37	2	2.10×10^−6^	Ser/Thr-kinase component of cyclin D-CDK4 complexes that regulate the cell-cycle during G(1)/S transition.
TOP2A	P11388	DNA topoisomerase 2-alpha	0.37	15	2.74×10^−74^	Control of topological states of DNA by transient breakage and subsequent rejoining of DNA strands.
CNTN1	Q12860	Contactin-1	0.40	10	1.36×10^−31^	Contactins mediate cell surface interactions during nervous system development.
AXL	P30530	Tyrosine-protein kinase receptor UFO	0.41	3	1.62×10^−6^	Receptor tyrosine kinase that transduces signals from the extracellular matrix into the cytoplasm.
SPINT2	O43291	Kunitz-type protease inhibitor 2	0.41	4	7.73×10^−5^	Inhibitor of HGF activator. Also inhibits plasmin, plasma and tissue kallikrein, and factor XIa.
GLB1	P16278	Beta-galactosidase	0.41	2	1.39×10^−4^	Isoform 2 has no beta-galactosidase catalytic activity, but plays functional roles in the formation of extracellular elastic fibers.
NRP1	O14786	Neuropilin-1	0.41	4	4.14×10^−15^	The soluble isoform 2 binds VEGF-165 and inhibits its binding to cells. It may also induce apoptosis by sequestering VEGF-165.
NUP210	Q8TEM1	Nuclear pore membrane glycoprotein 210	0.42	2	5.3×10^−4^	Nucleoporin essential for nuclear pore assembly and fusion, nuclear pore spacing, as well as structural integrity.
ATP6AP2	O75787	Renin receptor	0.42	3	6.11×10^−4^	Functions as a renin and prorenin cellular receptor. May mediate renin-dependent cellular responses by activating ERK1 and ERK2.
ASPH	Q12797	Aspartyl/asparaginyl beta-hydroxylase	0.42	28	2.50×10^−121^	Isoform 8: membrane-bound Ca(2+)-sensing protein, which is a structural component of the ER-plasma membrane junctions.
TGFBI	Q15582	Transforming growth factor-beta-induced protein ig-h3	0.43	4	1.36×10^−9^	Binds to type I, II, and IV collagens. This adhesion protein may play an important role in cell-collagen interactions.
TMTC3	Q6ZXV5	Transmembrane and TPR repeat-containing protein 3	0.43	2	8.9×10^−4^	Multi-pass membrane protein.
HSPD1	B9VP24	60 kDa chaperonin	0.44	2	2.56×10^−4^	ATP-binding
GOLM1	Q8NBJ4	Golgi membrane protein 1	0.44	3	1.56×10^−7^	Unknown. Cellular response protein to viral infection.
LMNB1	P20700	Lamin-B1	0.45	3	2.33×10^−32^	Component of the nuclear lamina, a fibrous layer on the nucleoplasmic side of the inner nuclear membrane.
DLGAP5	Q15398	Disks large-associated protein 5	0.45	4	6.29×10^−9^	Potential cell cycle regulator that may play a role in carcinogenesis of cancer cells.
RACGAP1	Q9H0H5	Rac GTPase-activating protein 1	0.45	2	1.68×10^−7^	Component of the centralspindlin complex that is required for myosin contractile ring formation during the cell cycle cytokinesis.
ANLN	Q9NQW6	Actin-binding protein anillin	0.46	2	2.64×10^−6^	Required for cytokinesis. Essential for the structural integrity of the cleavage furrow and for completion of cleavage furrow ingression.
CPSF2	Q9P2I0	Cleavage and polyadenylation specificity factor subunit 2	0.46	4	3.81×10^−4^	Component of the cleavage and polyadenylation specificity factor (CPSF) complex that play a key role in pre-mRNA 3′-end formation.
NDUFB11	Q9NX14	NADH dehydrogenase [ubiquinone] 1 beta subcomplex subunit 11, mitochondrial	0.46	2	8.19×10^−20^	Accessory subunit of the mitochondrial membrane respiratory chain NADH dehydrogenase.
DNAJB12	Q9NXW2	DnaJ homolog subfamily B member 12	0.46	2	1.87×10^−5^	Single-pass membrane protein
LONP2	Q86WA8	Lon protease homolog 2, peroxisomal	0.47	2	2.67×10^−2^	ATP-dependent serine protease that mediates the degradation of misfolded and unassembled polypeptides in the peroxisomal matrix.
TMEM165	Q9HC07	Transmembrane protein 165	0.47	2	1.35×10^−2^	May function as a calcium/proton transporter involved in calcium and in lysosomal pH homeostasis.
CASKIN2	Q8WXE0	Caskin-2	0.48	3	1.98×10^−7^	Unknown
BAIAP2	Q9UQB8	Brain-specific angiogenesis inhibitor 1-associated protein 2	0.48	3	5.12×10^−4^	Adapter protein that links membrane-bound small G-proteins to cytoplasmic effector proteins.
VMA21	Q3ZAQ7	Vacuolar ATPase assembly integral membrane protein VMA21	0.48	2	6.57×10^−4^	Required for the assembly of the V0 complex of the vacuolar ATPase (V-ATPase) in the endoplasmic reticulum.
PRAF2	O60831	PRA1 family protein 2	0.48	3	7.02×10^−14^	May be involved in ER/Golgi transport and vesicular traffic. Plays a proapoptic role in cerulenin-induced neuroblastoma apoptosis.
C1GALT1C1	Q96EU7	C1GALT1-specific chaperone 1	0.48	2	2.68×10^−3^	Probable chaperone required for the generation of 1 O-glycan Gal-beta1-3GalNAc-alpha1-Ser/Thr (T antigen).
FADS1	O60427	Fatty acid desaturase 1	0.49	2	7.08×10^−7^	Catalyzes biosynthesis of highly unsaturated fatty acids from precursor essential polyunsaturated fatty acids.
THNSL1	Q8IYQ7	Threonine synthase-like 1	0.50	2	1.36×10^−9^	Unknown
FADS2	O95864	Fatty acid desaturase 2	0.50	3	5.42×10^−5^	Catalyzes biosynthesis of highly unsaturated fatty acids from precursor essential polyunsaturated fatty acids.
SLMAP	Q14BN4	Sarcolemmal membrane-associated protein	0.50	2	2.97×10^−2^	May play a role during myoblast fusion.
AGFG2	O95081	Arf-GAP domain and FG repeat-containing protein 2	0.50	2	1.20×10^−2^	Has ARF GTPase activator activity and binds zinc.
UBE2S	Q16763	Ubiquitin-conjugating enzyme E2 S	0.50	6	7.55×10^−68^	Accepts ubiquitin from the E1 complex and catalyzes its covalent attachment to other proteins.

a Shown is the gene name, Uniprot accession number, protein ratio in DENV-2 infected cells compared to mock infected cells, the number of peptides used for quantification, the PEP score and a description of the protein function).

**Table 2:** Nuclear proteins increased or decreased in amount >2 fold during DENV-2 infection.

Gene Name^a^	UniprotID	Protein Name	DV2/Mock	Peps	PEP	Function
**Proteins increased >2fold in DENV-2 infected cells n = 13**						
EEF1A2	Q05639	Elongation factor 1-alpha 2	15.38	2	1.40×10^−106^	Promotes the GTP-dependent binding of aminoacyl-tRNA to the A-site of ribosomes during protein biosynthesis
JUN	P05412	Transcription factor AP-1	7.64	2	1.94×10^−15^	Transcription factor that recognizes and binds to the enhancer heptamer motif 5′-TGA[CG]TCA-3′
DHRS1	Q96LJ7	Dehydrogenase/reductase SDR family member 1	7.57	2	8.24×10^−4^	Belongs to the short-chain dehydrogenases/reductases (SDR) family.
EIF3D	O15371	Eukaryotic translation initiation factor 3 subunit D	7.05	2	6.16×10^−9^	Component of the eukaryotic translation initiation factor 3 (eIF-3) complex required for the initiation of protein synthesis.
SEC16	O15027	Protein transport protein Sec16A	5.83	2	1.21×10^−4^	Defines endoplasmic reticulum exit sites and is required for secretory cargo traffic from the ER to the Golgi apparatus.
HSPA5	P11021	78 kDa glucose-regulated protein	2.74	100	0	Probably plays a role in facilitating the assembly of multimeric protein complexes inside the ER.
SRPR	P08240	Signal recognition particle receptor subunit alpha	2.55	8	8.86×10^−42^	Ensures, in conjunction with the signal recognition particle, the correct targeting of the nascent secretory proteins to the ER.
SRP72	O76094	Signal recognition particle 72 kDa protein	2.34	9	3.96×10^−23^	Signal-recognition-particle assembly has a crucial role in targeting secretory proteins to the rough endoplasmic reticulum membrane.
HYOU1	Q9Y4L1	Hypoxia up-regulated protein 1	2.28	9	5.20×10^−63^	Pivotal role in cytoprotective mechanisms triggered by oxygen deprivation. Molecular chaperone involved in protein folding.
EIF3B	P55884	Eukaryotic translation initiation factor 3 subunit B	2.17	2	1.83×10^−4^	Component of the eukaryotic translation initiation factor 3 (eIF-3) complex required for the initiation of protein synthesis.
RELA	Q04206	Transcription factor p65	2.10	2	1.25×10^−3^	Nuclear factor NF-κB p65 subunit. NF-κB is a pleiotropic transcription factor involved in many biological processes.
SRP54	P61011	Signal recognition particle 54 kDa protein	2.06	4	3.44×10^−14^	Binds to the signal sequence of presecretory protein when they emerge from the ribosomes and transfers them to TRAM.
HSPA13	P48723	Heat shock 70 kDa protein 13	2.06	2	8.7×10^−3^	Belongs to the heat shock protein 70 family. Has peptide-independent ATPase activity.
**Proteins decreased >2fold in DENV-2 infected cells n = 44**						
MOGS	Q13724	Mannosyl-oligosaccharide glucosidase	0.09	2	1.46×10^−4^	Cleaves the distal alpha 1,2-linked glucose residue from the Glc(3)Man(9)GlcNAc(2) oligosaccharide precursor.
LYZ	P61626	Lysozyme C	0.11	2	8.24×10^−3^	Associated with the monocyte-macrophage system and enhance the activity of immunoagents. Anti-bacterial function.
RAB8B	Q92930	Ras-related protein Rab-8B	0.16	2	7.63×10^−104^	May be involved in vesicular trafficking and neurotransmitter release.
ELF3	P78545	ETS-related transcription factor Elf-3	0.27	2	1.53×10^−7^	Transcriptional activator that binds and transactivates sequences containing the consensus nucleotide core sequence GGA[AT].
BRD8	Q9H0E9	Bromodomain-containing protein 8	0.31	2	2.97×10^−3^	May act as a coactivator during transcriptional activation by hormone-activated nuclear receptors.
KIF20A	O95235	Kinesin-like protein KIF20A	0.34	2	1.72×10^−29^	Mitotic kinesin required for chromosome passenger complex-mediated cytokinesis.
ZMYM3	Q14202	Zinc finger MYM-type protein 3	0.35	2	3.08×10^−6^	Plays a role in the regulation of cell morphology and cytoskeletal organization.
CENPF	P49454	Centromere protein F	0.35	2	1.1678×10^−2^	Required for kinetochore function and chromosome segregation in mitosis.
PLK1	P53350	Serine/threonine-protein kinase PLK1	0.36	7	3.09×10^−17^	Serine/threonine-protein kinase that performs several important functions throughout M phase of the cell cycle.
CIZ1	Q9ULV3	Cip1-interacting zinc finger protein	0.36	2	7.19×10^−14^	May regulate the subcellular localization of CIP/WAF1.
SCD	O00767	Acyl-CoA desaturase	0.37	4	7.93×10^−4^	Component of the liver microsomal stearyl-CoA desaturase system, that catalyzes double bond formation into fatty acyl-CoA substrates.
PLP2	Q04941	Proteolipid protein 2	0.37	2	1.68×10^−4^	May play a role in cell differentiation in the intestinal epithelium.
NUSAP1	Q9BXS6	Nucleolar and spindle-associated protein 1	0.37	2	2.02×10^−5^	Microtubule-associated protein with the capacity to bundle and stabilize microtubules.
CDCA2	Q69YH5	Cell division cycle-associated protein 2	0.37	3	1.06×10^−58^	Regulator of chromosome structure during mitosis. Retains the architecture of condensin-depleted chromosomes through anaphase.
AURKB	Q96GD4	Aurora kinase B	0.38	7	1.67×10^−4^	Serine/threonine-protein kinase component of the chromosomal passenger complex, that acts as a key regulator of mitosis.
TGOLN2	O43493	Trans-Golgi network integral membrane protein 2	0.38	4	6.99×10^−12^	May be involved in regulating membrane traffic to and from trans-Golgi network.
KLF16	Q9BXK1	Krueppel-like factor 16	0.38	2	5.94×10^−22^	Transcription factor that binds GC and GT boxes and displaces Sp1 and Sp3 from these sequences.
RBM22	Q9NW64	Pre-mRNA-splicing factor RBM22	0.38	3	6.5×10^−4^	Involved in the first step of pre-mRNA splicing.
PITPNB	P48739	Phosphatidylinositol transfer protein beta isoform	0.38	2	5.01×10^−17^	Catalyzes the transfer of PtdIns and phosphatidylcholine between membranes
FAHD1	Q6P587	Acylpyruvase FAHD1, mitochondrial	0.38	2	2.13×10^−4^	Probable mitochondrial acylpyruvase which is able to hydrolyze acetylpyruvate and fumarylpyruvate in vitro.
TOR4A	Q9NXH8	Torsin-4A	0.39	2	6.61×10^−6^	Has ATP binding and nucleoside-triphosphatase activity.
ATF2	P15336	Cyclic AMP-dependent transcription factor ATF-2	0.39	2	4.42×10^−10^	Transcriptional activator, which binds to the cAMP-responsive element present in many viral and cellular promoters.
RAD51AP1	Q96B01	RAD51-associated protein 1	0.40	2	2.36×10^−4^	May participate in a DNA damage response pathway.
ATAD2	Q6PL18	ATPase family AAA domain-containing protein 2	0.41	5	8.85×10^−11^	May be a transcriptional coactivator of the nuclear receptor ESR1.
AURKA	O14965	Aurora kinase A	0.42	3	6.3×10^−5^	Mitotic serine/threonine kinases that contributes to the regulation of cell cycle progression.
CDCA8	Q53HL2	Borealin	0.43	7	1.18×10^−35^	Component of the chromosomal passenger complex, a complex that acts as a key regulator of mitosis.
CDCA5	Q96FF9	Sororin	0.44	2	1.26×10^−11^	Regulator of sister chromatid cohesion in mitosis stabilizing cohesin complex association with chromatin.
ECT2	Q9H8V3	Protein ECT2	0.44	4	6.77×10^−6^	Guanine nucleotide exchange factor (GEF) that catalyzes the exchange of GDP for GTP.
MKI67	P46013	Antigen KI-67	0.45	81	2.84×10^−301^	Thought to be required for maintaining cell proliferation.
TPX2	Q9ULW0	Targeting protein for Xklp2	0.45	11	1.66×10^−20^	Spindle assembly factor. Required for normal assembly of mitotic spindles.
RACGAP1	Q9H0H5	Rac GTPase-activating protein 1	0.45	3	1.83×10^−19^	Component of the centralspindlin complex that plays a role in signaling required for the myosin contractile ring formation.
POLR2D	O15514	DNA-directed RNA polymerase II subunit RPB4	0.45	2	7.86×10^−5^	DNA-dependent RNA polymerase catalyzes the transcription of DNA into RNA using the four ribonucleoside triphosphates.
MORF4L1	Q9UBU8	Mortality factor 4-like protein 1	0.46	5	1.11×10^−10^	Component of the NuA4 histone acetyltransferase complex which is involved in transcriptional activation of select genes.
ZCCHC8	Q6NZY4	Zinc finger CCHC domain-containing protein 8	0.46	2	2.26×10^−9^	May be involved in pre-mRNA splicing.
KIF23	Q02241	Kinesin-like protein KIF23	0.47	6	3.44×10^−18^	Component of the centralspindlin complex that plays a role in signaling required for the myosin contractile ring formation.
KIF20B	Q96Q89	Kinesin-like protein KIF20B	0.48	5	1.2×10^−5^	Plus-end-directed motor enzyme that is required for completion of cytokinesis.
RRP36	Q96EU6	Ribosomal RNA processing protein 36 homolog	0.48	4	4.65×10^−10^	Involved in the early processing steps of the pre-rRNA in the maturation pathway leading to the 18S rRNA.
CCNB1	P14635	G2/mitotic-specific cyclin-B1	0.48	3	8.5×10^−11^	Essential for the control of the cell cycle at the G2/M (mitosis) transition.
NTPCR	Q9BSD7	Cancer-related nucleoside-triphosphatase	0.48	2	2.33×10^−8^	Has nucleotide phosphatase activity towards ATP, GTP, CTP, TTP and UTP.
CDK2	P24941	Cyclin-dependent kinase 2	0.49	3	5.59×10^−17^	Serine/threonine-protein kinase involved in the control of the cell cycle; essential for meiosis, but dispensable for mitosis.
CREBBP	Q92793	CREB-binding protein	0.49	3	3.79×10^−6^	Acetylates histones, giving a specific tag for transcriptional activation and acetylates non-histone proteins, eg NCOA3/FOXO1.
TOP2A	P11388	DNA topoisomerase 2-alpha	0.50	91	0	Control of topological states of DNA by transient breakage and subsequent rejoining of DNA strands.
CNTN1	Q12860	Contactin-1	0.50	2	4.54×10^−5^	Contactins mediate cell surface interactions during nervous system development.
ATP1B3	P54709	Sodium/potassium-transporting ATPase subunit beta-3	0.50	4	7.4×10^−15^	Non-catalytic component of the active enzyme, which catalyzes the hydrolysis of ATP coupled with the exchange of Na(+) and K(+).

a Shown is the gene name, Uniprot accession number, protein ratio in DENV-2 infected cells compared to mock infected cells, the number of peptides used for quantification, the PEP score and a description of the protein function).

In previous studies that used 2D SDS-PAGE to analyze the proteome of DENV infected cells, between 350 [27] and 800 protein spots [24], [26] were detected, of which between 1 and 40 proteins were found to be significantly altered in amount in response to infection and subsequently identified by LC-MS/MS. The use of 2D difference gel electrophoresis (DIGE) for the analysis of West Nile virus (WNV) infected Vero cells extended the amount of protein spots detectable to ∼2000 and led to the reliable identification of 93 proteins that were significantly altered in amount [46]. A recent study that analyzed changes in the proteome of HeLa cells in response to infection with Japanese encephalitis virus by high throughput SILAC-MS analysis resulted in the identification and quantification of 978 and 1009 proteins in nuclear and cytoplasmic fractions of which 158 were significantly (>1.5 fold) altered in amount [47]. By contrast the use of high throughput SILAC-MS based protein quantification in this study resulted in the efficient quantification and identification of more than 3000 cellular proteins that neither changed nor significantly altered in amount during virus infection (listed in Table S1) and over 400 proteins that altered in amount by >1.5 fold. Similarly to the 2D SDS-PAGE proteomic studies [23]–[26], this study showed that DENV-2 infection results in a significant change in only a small subset of proteins. Although a number of the proteins that were found to be altered in amount during infection in this study were detected in other studies (described in more detail below) the majority of differentially regulated proteins detected in previous 2D SDS-PAGE based analyses of DENV infected cells were found to be unchanged in this analysis. This may reflect the different cell types, virus strains and time of infection analyzed in other studies, as previous proteomic studies have also observed both commonalities and differences in the alteration of proteins in different cell types at different times after infection [23], [24].

Analysis of the SILAC ratios of the proteins found in both the nuclear and cytoplasmic fractions showed that overall, proteins that were significantly decreased or increased in the cytoplasm were also decreased or increased respectively in the nucleus and vice versa (Figure 2C). There were 3 and 19 proteins that showed significant (>1.5 fold change) common increases or decreases in both the nuclear and cytoplasmic fractions respectively. The three proteins that increased; signal recognition particle receptor subunit α, 78 kDa glucose-regulated protein (also known as BiP) and hypoxia up-regulated protein 1 (HYOU1) are known to be ER associated, suggesting that their increase in the nuclear fraction may have been due to the association of peripheral ER membranes with the nucleus in infected cells. The 78 kDa glucose-regulated protein has previously been shown to be increased in DENV-2 infection of human K562 and monocyte derived dendritic cells and is required for the production of infectious virus [27]. Bioinformatic analysis showed that the proteins that commonly underwent significant decreases in the nuclear and cytoplasmic fractions were enriched in proteins involved in the cell cycle (see further discussion below). The proteins common to the nuclear and cytoplasmic fractions were analyzed to determine if there were proteins that significantly mislocalized during viral infection. Overall the analysis detected very few proteins that increased significantly (>1.5 fold) in one compartment and decreased significantly (>1.5 fold) in the other and vice versa. Only three proteins were found to significantly increase in the nucleus with a corresponding decrease in the cytoplasm, the p65 subunit of nuclear factor-κB (NF-κB) (RELA), ADP-ribosylation factor-like protein 6-interacting protein 1 (ARL6IP1) and Delta(3), delta(2)-enoyl-CoA isomerase 2 (ECI2). Nuclear translocation of NF-κB is well known to be involved in anti-viral defenses including against DENV [48] and the p65 subunit of NF-κB has previously been shown to nuclear localize during DENV infection [49], [50]. In contrast, ARL6IP1 and ECI2 are known to be associated with the ER and mitochondrial membranes respectively, suggesting that their accumulation in the nucleus was more likely due to an increased accumulation within perinuclear membranes associated with the viral replication complex. ARL6IP1 has been suggested to be involved in membrane trafficking [51] although its function is unknown. The enzyme fatty acid synthase (FASN) has been shown to be recruited to the site of DENV replication during infection, leading to increased fatty acid synthesis [52]. Similarly, ECI2 may also be redistributed in DENV infected cells. In contrast to FASN, ECI2 is involved in the degradation of fatty acids during fatty acid β-oxidation. Interestingly, proteomic and lipidomic analysis of hepatitis C virus (HCV) infected cells suggests that both lipid synthesis and metabolism is stimulated during infection and shown that Delta(3), delta(2)-enoyl-CoA isomerase 1 (DCI) is essential for the growth and RNA replication of HCV [53], [54]. No proteins were found to significantly decrease in the nucleus whilst increasing in the cytoplasm. Although it is likely that there was a greater redistribution of cellular proteins between the cytoplasm and nucleus during infection than revealed by this analysis, the amounts of these proteins or the timing of the changes did not allow their detection using our experimental system.

Analysis of the presence of the viral proteins in infected cells revealed that the C, prM, E, NS2A, NS3, NS4A and NS5 proteins could be detected in the cytoplasmic fraction from infected cells. These proteins and in addition the NS1 protein, could be detected in the nuclear fraction from infected cells. The small hydrophobic proteins NS2B and NS4B could not be detected in either fraction (Table 3). Although a number of DENV proteins (C, NS5, NS3) have been reported to detectable in the nucleus of infected cells [55]–[57] the E protein has not, suggesting that the intimate association of the DENV replication complex with perinuclear membranes, resulted in the enrichment of at least a proportion of the DENV proteins in the nuclear fraction.

**Table 3:** Viral proteins detected in infected A549 cells.

DENV-2 Protein	Cytoplasmic fraction		Nuclear fraction	
	peptide number^a^	% coverage^b^	peptide number	% coverage
C	2	8.8 (10/114)	2	17.5 (20/114)
pr	0	0 (0/91)	1	11 (10/91)
prM	3	17 (28/165)	4	23 (38/165)
M	2	36.5 (27/74)	2	36.5 (27/74)
E	13	30.1 (149/495)	14	26.9 (133/495)
NS1	0	0 (0/352)	2	5.7 (20/352)
NS2A	2	11.9 (26/218)	1	5.1 (11/218)
NS2B	0	0 (130)	0	0 (130)
NS3	35	57 (352/618)	39	54.4 (336/618)
NS4A	2	15 (19/127)	1	13.4 (17/127)
2K	0	0 (0/23)	0	0 (0/23)
NS4B	0	0 (0/248)	0	0 (0/248)
NS5	39	51 (459/900)	43	48.8 (439/900)

*^a^*number of individual peptides identified for each protein.

*^b^*the number of amino acids covered compared to the total number of residues per protein.

### Bioinformatic analysis

**Figure 3:**
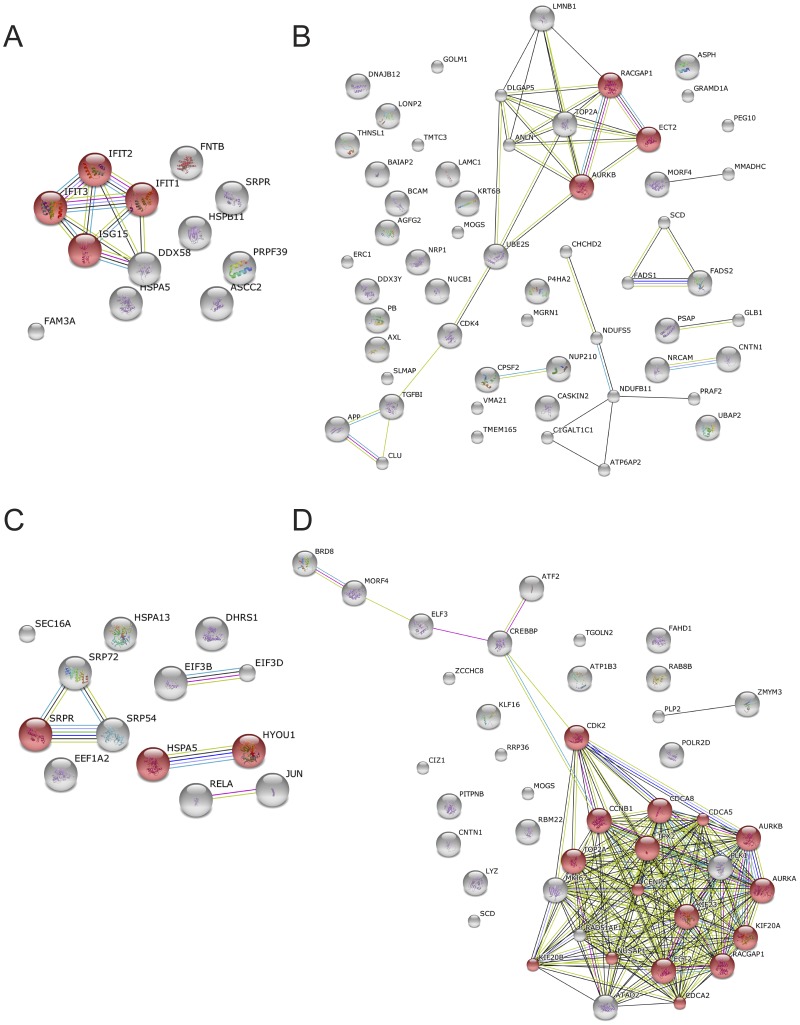
Protein association networks detected using the STRING database. Proteins that were altered in abundance either >2 or <2 fold respectively in the cytoplasmic (A) and (B) or nuclear fractions (C) and (D) from DENV-2 infected cells compared to mock infected cells were analyzed using STRING. The nodes shaded in red indicate proteins that were most significantly enriched for a specific GO Biological Process term in each STRING network displayed. For the cytoplasmic proteins that increased >2 and <2 fold in DENV-2 infected cells, the GO terms were “type I interferon-mediated signaling pathway” (*P* = 2.9×10^−3^) and “positive regulation of cell cytokinesis” (*P* = 4.8×10^−2^) respectively, whilst for the nuclear proteins that increased >2 and <2 fold in DENV-2 infected cells, the GO terms were “activation of signaling proteins involved in the unfolded protein response” (*P* = 4.4×10^−2^) and “cell division” (*P* = 5.2×10^−12^).

Gene ontology (GO) based enrichment analysis and protein network analysis was done for proteins that had SILAC quantification ratios that showed a ≥ 2 and ≥1.5 fold increase or decrease in the nuclear and cytoplasmic fractions from DENV-2 infected cells compared to mock infected cells (Tables 1 and 2, Table S1). DAVID [40], [41] was first used to assign biological functions to proteins that were significantly altered in amount. The biological functions were then clustered into functionally related groups and examined for significance of the protein-term enrichment using a modified Fisher's exact test (EASE score). The DAVID pathway viewer was also used to determine if specific cellular networks were enriched with proteins from the lists. The three most significantly enriched functionally related clusters and pathways that were significantly enriched are shown in Table 4 and discussed below. The STRING database was used to identify protein networks that may be modulated in response to infection (Figure 3). The results of these analyses are described as follows.

**Table 4:** DAVID analysis of proteins significantly altered in amount in DENV-2 infected cells.

	Nuclear^a^	Cytoplasmic
**>2 fold increase**	Signal recognition particle/ribonucleoprotein binding/protein export (3.44)	IFN induced tetratricopeptide repeat (2.99)
	Translation factor activity/nucleic acid binding (2.13)	
	Heat shock/ER (1.94)	
	Protein export (KEGG pathway; *p* value = 2.2×10^−5^)	RIG-I-like receptor signalling (KEGG pathway; *p* value = 4.1×10^−2^)
**>1.5 fold increase**	ER membrane/nuclear envelope-ER network (7.73)	IFN induced tetratricopeptide repeat (2.19)
	ER lumen/membrane-bound vesicle (5.68)	ER membrane/nuclear envelope-ER network (1.96)
	Co-translational protein targeting/protein folding (5.29)	
	Protein export (KEGG pathway; *p* value = 6.8×10^−8^)	Alanine, aspartate and glutamate metabolism (KEGG pathway; *p* value = 6.5×10^−2^)
**>2 fold decrease**	Cell cycle regulation/mitosis/M phase (8.97)	Electron transport/oxidation reduction (1.71)
	Nuclear lumen/nucleoplasm (5.95)	Oxidoreductase activity/biosynthesis of unsaturated fatty acids/fatty acid biosynthesis (1.62)
	Nucleotide/ATP/purine binding (3.75)	Cell morphogenesis/cell adhesion/extracellular matrix (1.44)
	Cell cycle (KEGG pathway; *p* value = 2.7×10^−3^)	Biosynthesis of unsaturated fatty acids (KEGG pathway; *p* value = 2.6×10^−3^)
**>1.5 fold decrease**	Nuclear lumen/nucleoplasm (15.77)	Cell division/cell cycle (2.45)
	Cell cycle regulation/mitosis/M phase (12.01)	Oxidoreductase activity/iron ion binding/(2.3)
	Chromosome/transcription/DNA binding (5.73)	ER/ER membrane (2.1)
	Cell cycle (KEGG pathway; p value = 5.2×10^−4^)	Steroid biosynthesis (KEGG pathway; *p* value = 6.2×10^−4^), Biosynthesis of unsaturated fatty acids (KEGG pathway; *p* value = 2.1×10^−2^) Lysosome (KEGG pathway; *p* value = 3.1×10^−2^)

*^a^*Proteins that showed a >2 or 1.5 fold change in abundance in DENV-2 infected A549 cells at 28 hours p.i. were analyzed for the presence of enriched groups of proteins and proteins involved in specific host pathways using DAVID. The functional descriptors for the 3 clusters of proteins that were most enriched for each category (with an EASE score greater than 1.3 (corresponding to a *p* value <0.05)) are shown with the corresponding EASE score. The two KEGG pathways that were most significantly enriched (with a *p* value <0.05) are also shown with the corresponding *p* value.

#### Proteins increased in the cytoplasmic fraction during infection

The proteins that increased by >2 fold in the cytoplasmic fraction (Table 1) were found to be significantly enriched in proteins involved in the type I interferon (IFN) response (Figure 3, Table 4). This is not surprising and in line with previous transcriptomic and cell based studies [7]–[12], [14]–[19], [58] showing that DENV infection induces the IFN response, although it is also capable of suppressing IFN production and signaling [59], [60]. Furthermore, analysis of JEV infected HeLa cells by SILAC-MS based proteomics also identified interferon regulated proteins as a major class of proteins upregulated in response to infection [47]. The three proteins IFIT-3, 1 and 2 showed the greatest increase in amount in the cytoplasmic fraction from infected cells. IFIT-3, 1 and 2 are *Ifit* family members that are induced in a variety of cell types in response to viral infection *via* interferon-dependent and independent signaling pathways [61] and capable of inhibiting viral replication by interfering with protein translation. Infection of immunocompetent adult mice with WNV led to increases in the transcript levels of the murine equivalents of IFIT-1, 2 and 3 in brain tissue and for IFIT-2, immunostaining revealed a corresponding increase in protein levels [62]. Flaviviruses appear to mimimize the inhibitory effects of IFIT proteins by 2′-O-methylation of the viral RNA cap structure [63], [64]. The murine IFIT-1 equivalent was found to play a dominant role in restricting infection of WNV lacking 2′-O-methylation [63] however overexpression of IFIT-1 in HEK293T cells did not inhibit DENV or WNV replication [65]. When cytoplasmic proteins that increased >1.5 fold during infection were also considered in the analysis, proteins associated with the additional annotation terms “ER/ER membrane” and “unfolded protein response” (STRING) were found to be enriched, supporting previous studies that have shown that the unfolded protein response is induced in response to DENV infection [66]–[69].

#### Proteins increased in the nuclear fraction during infection

Bioinformatic analysis of proteins that increased >2 and 1.5 fold in the nuclear fraction of DENV infected cells collectively showed proteins belonging to functional annotation clusters associated with the terms signal recognition particle/ribonucleoprotein binding/protein export; translation factor activity/nucleic acid binding; heat shock protein/ER and protein folding/the unfolded protein response. The strong association of these terms with the ER once again suggested that the increased amounts of proteins found in the nuclear fraction in response to viral infection was due to the close association of viral induced replication structures derived from the ER membranes with the nuclear membrane [4] rather than increased localization of these proteins into the nucleus. This is supported by the finding that elongation factor 1-α2, the protein most increased in amount in the nuclear fraction of infected cells, has previously been shown to interact with the DENV 3′ untranslated region and co-localize with the DENV replication complex [70], [71].

#### Proteins decreased in the cytoplasmic fraction during infection

Analysis of proteins that decreased >2 fold in the cytoplasmic fraction suggested that the processes of electron transport/oxidation-reduction, the biosynthesis of unsaturated fatty acids and cell morphogenesis were modulated during DENV infection. Previous studies have shown that DENV and other flaviviruses are able to manipulate lipid biosynthesis and redistribute lipids within cells to establish the viral replication complex and evade host defense mechanisms [72]. DENV infection is known to modulate fatty acid [52], [73] and cholesterol biosynthesis [74] and trigger autophagy, leading to alterations in cellular lipid metabolism [75]. A number of proteins including stearoyl–CoA desaturase (SCD), fatty acid desaturase 1 (FADS1) and 2 (FADS2) decreased during infection, although the level of FASN, previously shown to be recruited to the DENV replication complex and required for virus replication [52] remained unchanged (Table S1). SCD is a rate limiting enzyme in the synthesis of monounsaturated fatty acids. Mice deficient in SCD were found to have reduced triglyceride storage and increased lipid oxidation [76], [77]. Interestingly, DENV infection has also been shown to result in a depletion of lipid droplet triglyceride stores and increased lipid β-oxidation [73], [75]. Interestingly, analysis of JEV infected cells also showed that a number of proteins involved in cholesterol biosynthesis were down regulated [47]. Our results provide further evidence that flavivirus infection both increases and decreases specific lipid synthesis pathways to facilitate virus replication and identifies new targets for further investigation. Inclusion of cytoplasmic proteins that decreased in amount by >1.5 fold in the analysis, highlighted the enrichment of proteins involved in cell cycle regulation as discussed below.

#### Proteins decreased in the nuclear fraction during infection

The most striking finding of the bioinformatic analysis was the highly significant association of a large number of proteins that decreased in the nuclear fraction with functional annotation terms related to cell cycle regulation and in particular mitosis. Proteins that are known to play key roles in cell cycle regulation such as the cyclin dependent kinases 1 (CDK1), 2 (CDK2) and 4 (CDK4), cyclin B1 (CCNB1) and Aurora kinases A (AURKA) and B (AURKB) were decreased in the nuclear and cytoplasmic fractions, in addition to many other cell cycle associated proteins (Figure 3, Tables 1 and 3, Table S1). The analysis suggested that DENV infection is able to modulate the cell cycle and points to a blockage during the G2/M phase or during progression or exit from M phase. There are few reports describing the effects of DENV infection on the cell cycle. Infection of endothelial cells derived from human umbilical cord veins with DENV-1 to -4 was observed to result in endothelial cell proliferation and an increased number of mitotic cells [78]. It has been shown that increased amounts of virus were released from C6/36 mosquito cells but not human Huh-7 liver cells stalled during S phase, presumably due to increased virus assembly [79]. However, DENV-2 infection was not found to affect the cell cycle in either of these two cell types. A study examining the effect of the cell cycle on DENV replication showed that the effects may be cell type specific [80], which may explain the results of this study using DENV-2 infected A549 cells. For a number of other viruses it has been shown that viral replication arrests the cell cycle at the G2/M transition, including HCV [81]–[83]. In the case of HCV, specific virus gene products have been linked to the induction of cell cycle arrest which may also be the case for DENV and is an area for further investigation.

### Validation of the SILAC/MS results by Western blot and immunofluorescence assays

In order to validate the SILAC-MS analysis, the amounts of seven proteins that changed significantly in amount (≥1.5 fold increase or decrease) in DENV-2 compared to mock infected cells were examined by Western blot analysis, both in the original SILAC samples used for the MS analysis and in total cell lysates prepared from two human cell types (A549 and HEK293 cells) over a longer time course of DENV-2 infection. The proteins were selected based on i) the known relevance of the proteins to viral infection, ii) the magnitude of the increase/decrease in protein amount and iii) an example of a protein participating in a specific cellular pathway or process (as determined by gene enrichment and pathways analysis). Overall we focused on proteins that decreased in the nucleus and/or cytoplasm of infected cells as such proteins may be actively degraded during viral infection due to their role in anti-viral processes. The properties of the selected proteins and their SILAC ratios in DENV-2 compared to mock infected cells are shown in Table 5 and a brief summary of their function/s can be described as follows. Cathepsin L1 (CTSL1) is a lysosomal cysteine proteinase that is primarily involved in protein degradation and processing antigen for MHC class II presentation [84]. CTSL1 is also recruited to the nucleus in mammalian cells where it is known to cleave transcription factors and the tail of histone H3 [85]. ELKS/Rab6-interacting/CAST family member 1 (ERC1) has been shown to regulate the activation of NF-kB [86] and trafficking of Ras-related proteins Rab-6A and Rab8A from the Golgi to the endosome [87], [88], both of which are potentially important in DENV infection. Importin-subunit α2 (KPNA2) is involved in the nuclear transport of proteins. Importin-α is an adaptor protein that binds to a nuclear localization sequence on a cargo protein and in turn binds to importin-β to form a complex that is transported through the nuclear pore complex [89]. There are seven human isoforms of importin-α [90]. Although KPNA1, 3, 4 and 5 were also detected in the analysis, only KPNA2 was found to be significantly decreased in both the cytoplasmic and nuclear fractions of DENV-2 infected cells. The murine counterpart of KPNA2 has been previously been shown to bind to the DENV-2 NS5 protein which accumulates in the nucleus during infection [43], [55]. Mitofusin 1 (MFN1) is a GTPase that is localized to the mitochondrial outer membrane and regulates mitochondrial fusion [91]. A number of reports have implicated mitochondrial fusion dynamics and MFN1 in RIG-I mediated signaling and shown that MFN1 interacts with mitochondrial antiviral-signaling protein, suggesting MFN1 plays a role in the cellular anti-viral response [92], [93]. PRA1 family protein 2 (PRAF2) is an integral membrane protein with four-transmembrane domains that is localized to the ER and *trans*-Golgi network and is believed to play a regulatory role in vesicular trafficking [94], [95]. PRAF2 is also known to interact with C-C chemokine receptor type 5 (CCR5) [94], [96] and Bcl-xL to modulate cell survival [96]. Ubiquitin-conjugating enzyme E2 S (UBE2S) functions as an auxiliary factor in the multi-subunit anaphase-promoting complex (APC), a cell cycle-regulated E3 ubiquitin ligase that controls progression through mitosis [97]. A number of viruses are now known to modulate the activity of the APC, including through the targeting of APC subunits for degradation, to facilitate their replication [98]. Hypoxia up-regulated protein 1 (HYOU1) is an inducible ER chaperone protein that is upregulated in response to cellular stresses including the unfolded protein response and hypoxia. HYOU1 is known to be cytoprotective and is involved in anti-apoptotic signaling mechanisms [99], [100].

**Table 5:** Properties of the proteins selected for biological validation.

Gene Name^a^	Uniprot ID	Protein Name	MW (kDa)	DV2/Mock	Sequence coverage %	Peps	PEP
**CTSL1**	P07711	Cathepsin L1	37.5	0.64	8.4	3	7.03×10^−8^
				NaN^b^		1	6.87×10^−4^
**ERC1**	Q8IUD2	ELKS/Rab6-interacting/CAST family member 1	128.1	0.096	5.5	4	4.01×10^−20^
				ND^c^			
**KPNA2**	P52292	Importin subunit alpha-2	57.9	0.56	39.7	23	0
				0.61	14.2	7	3.04×10^−108^
**MFN1**	Q8IWA4	Mitofusin-1	87	0.58	2.7	2	2.57×10^−2^
				ND			
**PRAF2**	O60831	PRA1 family protein 2	19.3	0.48	12.4	3	7.03×10^−14^
				NaN		1	7.15×10^−3^
**UBE2S**	Q16763	Ubiquitin-conjugating enzyme E2 S	23.8	0.50	42.8	6	7.55×10^−68^
				ND			
**HYOU1**	Q9Y4L1	Hypoxia up-regulated protein 1	111.3	1.64	22.7	24	1.54×10^−100^
				2.28	7.9	9	5.20×10^−63^

a Shown is the gene name, Uniprot accession number, protein name and molecular mass, protein ratio in DENV-2 infected cells compared to mock infected cells, the peptide sequence coverage and number of peptides used for quantification, and the PEP score.

b NaN, one or more peptides corresponding to the proteins were identified but they were not used for quantitation,

c ND, no peptides corresponding to the protein were identified.

**Figure 4:**
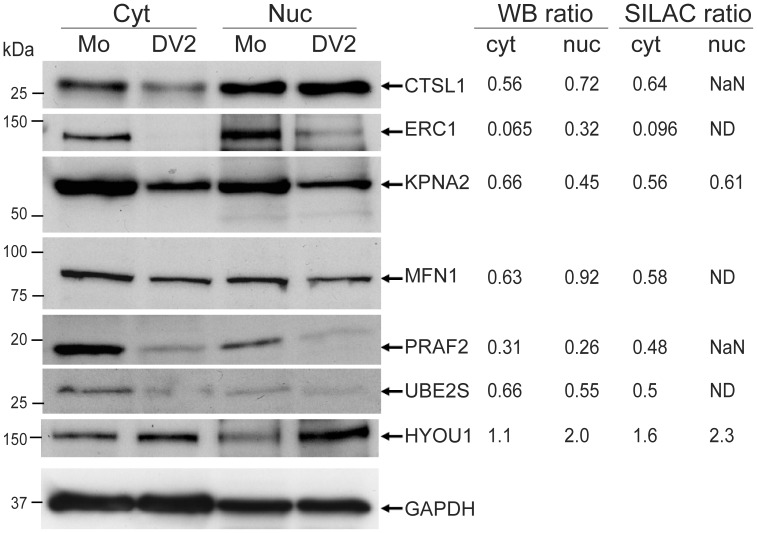
Validation of the SILAC/MS results by Western blot analysis. SILAC labeled A549 cells that had been either DENV-2 (DV2) or mock (Mo) infected were harvested at 24 hours p.i. and used for the production of cytoplasmic (Cyt) and nuclear (Nuc) extracts that were subsequently used for MS analysis. The levels of the cellular proteins CTSL1, ERC1, KPNA2, MFN1, PRAF2, UBE2S, HYOU1 and GAPDH (used as a loading control) in each fraction (ten μg of protein loaded per lane) were analyzed by Western blotting using specific anti-sera. The results shown are typical of two independent Western blotting experiments. The positions of relevant molecular mass markers are shown in kDa. The intensity of the bands was determined using ImageJ and normalized to the intensity of the GAPDH bands in each experiment. The average values of the intensities were used to determine the Western blot ratios (DV2/Mo) of each protein in the cytoplasmic and nuclear fractions. NaN  =  one or more peptides corresponding to the proteins were identified but they were not used for quantitation; ND  =  no peptides corresponding to the protein were identified.

Initially the amounts of the seven proteins were analyzed in the SILAC labeled cytoplasmic and nuclear fractions used for the MS/MS analysis (Figure 4). In agreement with the MS/MS analysis, there was an observable decrease in the amounts of the proteins CTSL1, ERC1, KPNA2, MFN1, PRAF2 and UBE2S in the cytoplasmic fraction from DENV-2 infected cells whereas there was an increase in HYOU1. The most dramatic decreases were observed for the proteins ERC1 and PRAF2 that correlated with the quantitative MS/MS analysis. MS/MS analysis of the nuclear fractions from DENV-2 and mock infected cells resulted in the quantification of peptides corresponding to KPNA2 and HYOU1, that decreased and increased in infected cells respectively, which was confirmed by Western blot analysis. Although peptides corresponding to CTSL1, PRAF2 and UBE2S were identified in the tryptic digests from the nuclear fraction, they were not used for quantification. However all of these proteins were detected in the nuclear fractions by Western blot analysis at higher levels than suggested by the SILAC-MS analysis (Figure 4). Their abundance in the nuclear faction of DENV-2 infected cells, compared to mock infected cells reflected the changes in protein amounts observed in the cytoplasmic fraction. The levels of GAPDH were examined in the cellular extracts as a loading control as the MS/MS analysis showed that the level of GAPDH was essentially the same in the cytoplasm and nucleus of DENV-2 and mock infected cells.

**Figure 5:**
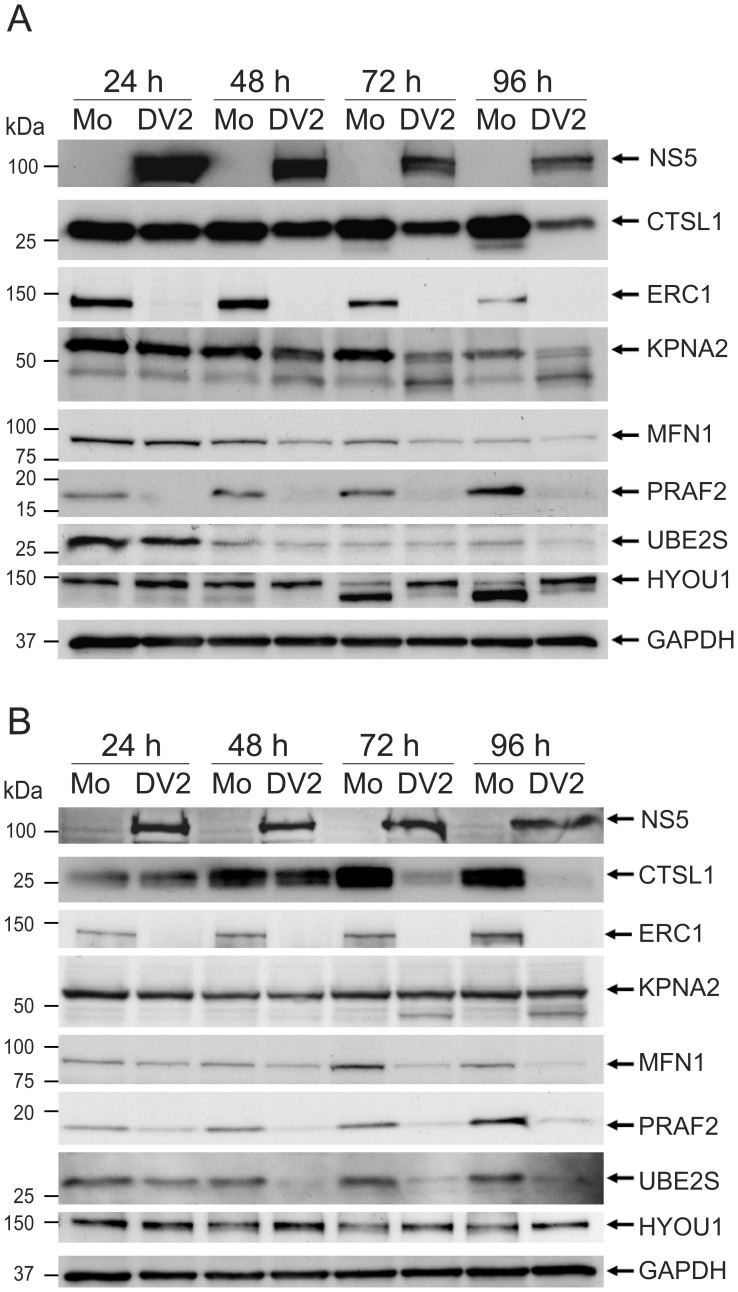
Validation of the SILAC/MS results over a time course of infection by Western blot analysis. (A) A549 cells and (B) HEK-293 cells were infected with DENV-2 (DV2) at a m.o.i. of 3 or mock (Mo) infected. At the indicated times p.i. (24, 48, 72 and 96 h) the cells were harvested and lysed in 2X SDS-PAGE sample buffer to produce a total cell lysate. The levels of the viral NS5 protein and the cellular proteins CTSL1, ERC1, KPNA2, MFN1, PRAF2, UBE2S, HYOU1 and GAPDH (used as a loading control) in each lysate (ten μg of protein loaded per lane) were analyzed by Western blotting using specific anti-sera. The results shown are typical of at least two independent Western blotting experiments. The positions of relevant molecular mass markers are shown in kDa.

Following the validation of the proteomic analysis for the selected proteins, using the SILAC samples used for MS/MS analysis, it was of interest to examine whether the amounts of the proteins were consistently altered over a time course of DENV-2 infection and in another cell type. The amounts of the selected proteins were therefore examined in human A549 and HEK-293 cells at a number of time points after infection with DENV-2, in comparison to mock infected cells. Cell samples were harvested at 24, 48, 72, and 96 hours p.i. and used to produce total cell lysates which were examined by Western blot analysis (Figures 5A and 5B). To ensure that the respective cell lines were infected with DENV-2, the amount of the DENV-2 NS5 protein in the samples was examined, confirming the infections. The seven selected cellular proteins were all detected in mock infected A549 and HEK-293 cells over the time course of infection. In support of the SILAC-MS analysis, all of the proteins were altered in amount in one or both of the A549 and HEK-293 cell lines over the time course of infection. As observed using the SILAC labeled cellular fractions, the most dramatic changes occurred in the amounts of the ERC1 and PRAF2 proteins. At all time points over the infection time course, ERC1 was reduced to almost undetectable levels in infected A549 and HEK-293 cells compared to the mock infected cells. The amount of PRAF2 increased in the mock infected cells over time. By contrast, the amount of PRAF2 in DENV-2 infected A549 and HEK293 cells was severely decreased over the infection time course, with the greatest reduction observed in A549 cells to almost undetectable levels. The amounts of CTSL1 and MFN1 also decreased in both DENV-2 infected cell types compared to the mock infected cells as the infection progressed. For CSTL1, the decrease was continual in A549 cells whereas in HEK-293 cells, the change in CSTL1 amount was less apparent in infected cells at 24 hours p.i. but decreased more severely at later times in the infection (72 and 96 hours p.i.). Although the amounts of MFN1 varied over the infection time course, both in mock and DENV-2 infected A549 and HEK-293 cells, the amounts of MFN1 in DENV-2 infected cells was consistently decreased at all time points over the infection time course. Compared to the result from the SILAC-MS analysis, the amounts of UBE2S appeared similar in DENV-2 and mock infected A549 cells over the infection time course. However, in HEK-293 infected cells, there was a clear decline in UBE2S amount from 24 hours p.i. and the protein was significantly decreased in amount from 48–96 hours p.i. In comparison to the proteins described above, KPNA2 was less decreased in DENV-2 infected A549 and HEK-293 cells compared to mock infected cells. In A549 cells, the level of KPNA2 was decreased more substantially from 48 hours p.i. onwards. In addition, the amounts of a lower molecular weight protein (∼45 kDa) appeared to increase as the level of KPNA2 decreased. In HEK-293 cells, the decrease in KPNA2 in DENV-2 infected cells was not clearly evident, although a protein corresponding in size to that observed in the A549 cell lysates also increased in amount as the infection progressed (72 and 96 hours p.i.). As observed in the SILAC-MS analysis, the amounts of HYOU1 increased in infected cells over the infection time course. For the A549 cells, the amounts of HYOU1 were not observably increased at 24 and 48 hours p.i. However at 72–96 h post-infection, the amount of HYOU1in the mock infected cells was severely decreased with a corresponding increase in the amounts of a protein of ∼100 kDa. In DENV-2 infected HEK-293 cells there was a consistent increase in the amount of HYOU1 compared to mock infected cells at all times post-infection. Overall the experiments confirmed the results of the SILAC-MS analysis and identified seven proteins that altered in amount in one or more cell types during DENV-2 infection.

**Figure 6:**
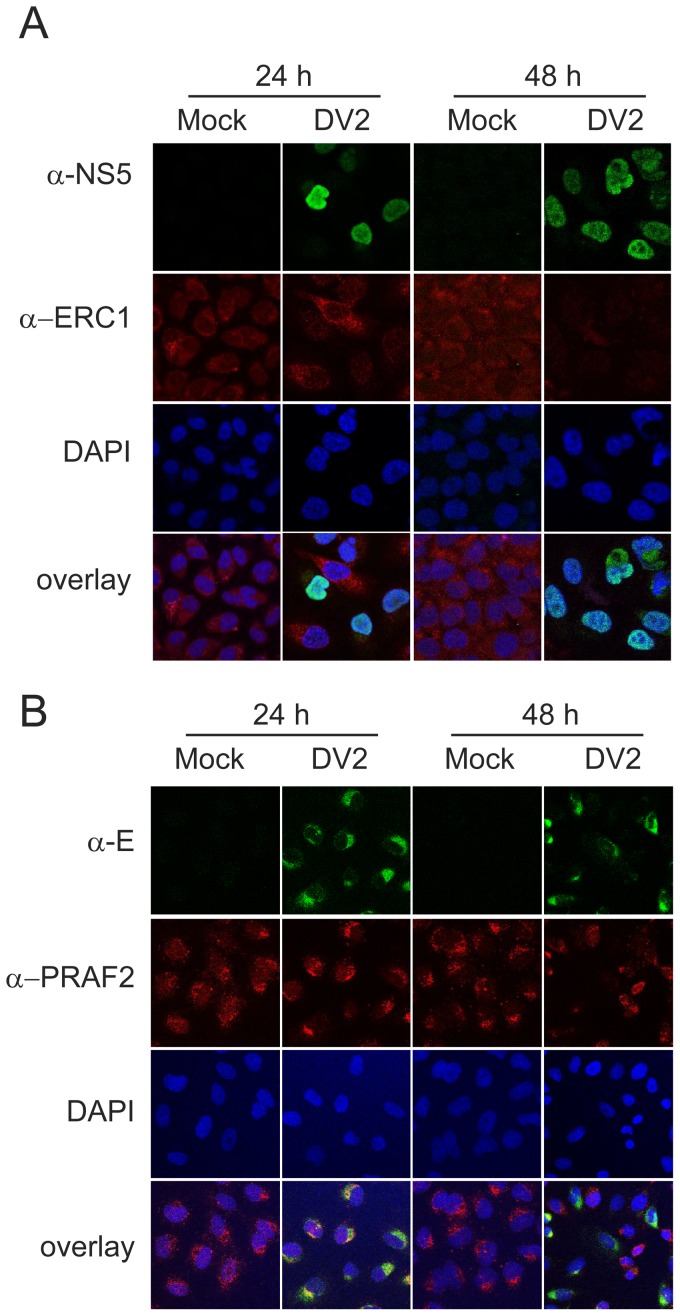
Validation of the SILAC/MS analysis for the ERC1 and PRAF2 proteins by IFA. Confocal laser scanning images of (A) A549 cells infected with DENV-2 (DV2) at a m.o.i. of 1 or mock infected and immunostained with anti-NS5 (α-NS5) and anti-ERC1 (α-ERC1) antibodies at 24 hours p.i. and 48 hours p.i. (B) A549 cells infected with DENV-2 (DV2) at a m.o.i. of 1 and immunostained with anti-E (α-E) and anti-PRAF2 (α-PRAF2) antibodies at 24 hours p.i. and 48 hours p.i. The results shown are typical of at least two independent infection and IFA experiments. Nuclear DNA was stained with DAPI.

To complement the Western blot analysis, the amounts and localization of ERC1 and PRAF2, that were most decreased in DENV-2 infected cells, were analyzed by IFA. A549 cells were infected with DENV-2 at a m.o.i. of 1 or mock infected. At 24 and 48 hours p.i. the cells were examined for the presence of ERC1, PRAF2 and either the DENV-2 E or NS5 proteins by IFA (Figures 6A and 6B). ERC1 was predominantly localized to the cytoplasm in mock infected cells. By 24 hours p.i. the amount of ERC1 was observably diminished in DENV-2 infected cells and by 48 hours p.i. was barely detectable (Figure 6A). PRAF2 accumulated most strongly in the cytoplasm of the mock infected cells, showing a punctate distribution, supporting the previous observation that the protein was localized to the ER and trans-Golgi network [94]. By contrast, in DENV-2 infected cells, PRAF2 appeared to co-localize with the viral E protein at 24 hours p.i. and in the majority of infected cells was severely decreased in amount by 48 hours p.i. (Figure 6B). In a similar fashion, the levels of CSTL1, MFN1, KPNA2 and UBE2S in DENV-2 infected were examined by IFA. However for CSTL1, MFN1 and UBE2S, the specific antibodies available (that had been used for Western blot analysis) could either not detect the proteins of interest or reacted non-specifically with other proteins (data not shown). For KPNA2, there appeared to be no difference in the amount or localization during DENV-2 infection compared to mock infected cells at 24 and 48 hours p.i. (data not shown). This may have been due to an interaction of the anti-KPNA2 antisera with the smaller product detected in the Western blot analysis of KPNA2, masking any overall decrease in KPNA2 amounts. The analysis supported the Western blot analysis in that proteins that were observed to be significantly decreased during infection by Western blot analysis were also observed to decrease in virus infected cells when examined by IFA. Proteins that showed a smaller decrease were found to be more difficult to visualize by IFA although this was also dependent on the availability of highly specific antibodies.

The biological validation identified two proteins that were severely decreased in DENV-2 infected cells, ERC1 and PRAF2. The decrease in the amounts of these proteins may be a direct or indirect effect of virus replication. ERC1 has previously been identified to interact with the DENV NS5 protein in a yeast two hybrid screen [20] which we have also observed using a high-throughput co-immunoprecipitation analysis (Hannemann *et al*., manuscript in preparation). siRNA knockdown of ERC1 inhibited the replication of a DENV replicon [20] suggesting that ERC1 is required for efficient DENV replication which appears to contradict this study showing that ERC1 is decreased during DENV infection. However it may be that ERC1 plays different roles at various stages of the DENV lifecycle which illustrates how the use of different high-throughput approaches can complement one another to increase our understanding of the role of cellular proteins in the DENV lifecycle.

## Conclusions

The results of this study demonstrate for the first time the power of quantitative LC-MS/MS for investigating the host cell response to DENV infection. Compared to traditional 2D-PAGE based analysis, thousands of proteins can be both identified and quantified in response to DENV infection. Proteins that are either significantly altered or remain unchanged in amount during infection were identified, providing a resource for further bioinformatic analyses and biological validation. Using this approach we identified specific host proteins and cellular processes that are modulated during DENV infection. A number of the cellular processes identified have been associated with DENV infection in previous transcriptomic and siRNA studies, validating our results, however others were novel and require further investigation. In addition, changes in the amounts of specific proteins that were not associated with well studied pathways were identified and validated, and these proteins can now be assessed for their role in the DENV lifecycle. The use of high-throughput quantitative proteomic analysis complements other high-throughput approaches, and in combination offers the possibility to identify cellular proteins that may be targets against which novel anti-viral strategies can be developed which are urgently needed against this important disease.

## Supporting Information

Table S1 **List of the proteins identified and quantified by SILAC LC-MS/MS in cytoplasmic and nuclear fractions from DENV-2 infected A549 cells.**
(XLS)
